# Cherry Extract from *Prunus avium* L. to Improve the Resistance of Endothelial Cells to Oxidative Stress: Mucoadhesive Chitosan vs. Poly(lactic-*co*-glycolic acid) Nanoparticles

**DOI:** 10.3390/ijms20071759

**Published:** 2019-04-10

**Authors:** Denise Beconcini, Angela Fabiano, Rossella Di Stefano, Maria Helena Macedo, Francesca Felice, Ylenia Zambito, Bruno Sarmento

**Affiliations:** 1 Department of Life Sciences, University of Siena, via P.A. Mattioli 4, 53100 Siena, Italy; denisebeconcini@gmail.com; 2 Department of Pharmacy, University of Pisa, via Bonanno 33, 56100 Pisa, Italy; angela.fabiano@unipi.it; 3 Cardiovascular Research Laboratory, Department of Surgery, Medical, Molecular, and Critical Area Pathology, University of Pisa, via Paradisa 2, 56100 Pisa, Italy; rossella.distefano@unipi.it (R.D.S.); francesca.felice77@hotmail.it (F.F.); 4 Interdepartmental Research Center Nutraceuticals and Food for Health, University of Pisa, via Borghetto 80, 56100 Pisa, Italy; 5 i3S-Instituto de Investigação e Inovação em Saúde, University of Porto, Rua Alfredo Allen 208, 4200-153 Porto, Portugal; helena.macedo@i3s.up.pt (M.H.M.); bruno.sarmento@ineb.up.pt (B.S.); 6 INEB—Instituto de Engenharia Biomédica, Universidade do Porto, Rua Alfredo Allen, 208, 4200-135 Porto, Portugal; 7 ICBAS—Instituto de Ciências Biomédicas Abel Salazar, University of Porto, Rua de Jorge Viterbo Ferreira 228, 4050-313 Porto, Portugal; 8 CESPU, Instituto de Investigação e Formação Avançada em Ciências e Tecnologias da Saúde, Rua Central de Gandra, 1317, 4585-116 Gandra, Portugal

**Keywords:** sweet cherry (*Prunus avium* L.), polyphenols, chitosan nanoparticles, PLGA nanoparticles, intestinal permeability, oxidative stress, HUVECs

## Abstract

Polyphenolic compounds contained in cherry extract (CE) are well known for their antioxidant and anti-inflammatory properties. Unfortunately, most of these natural compounds have low oral bioavailability, reducing their widespread use. Here, different concentrations of polyphenol-rich CE from Tuscany (Italy), encapsulated in poly(lactic-*co*-glycolic acid) (PLGA) nanoparticles (NPs), were compared with those encapsulated in two NP types, different from each other in terms of mucoadhesivity, obtained with chitosan derivatives (Ch-der), regarding CE gastrointestinal (GI) permeability and protective effect on oxidative stress. Different NP systems were physico-chemically characterized, and the antioxidant GI permeability was evaluated in a triple-cell co-culture model (Caco-2/HT29-MTX/Raji B), resembling the intestine. PLGA NPs efficiently entrapped CE (up to 840 µg gallic acid equivalent (GAE)/mL) without altering size (210 nm), polydispersity index (0.05), or zeta potential (−10.7 mV). Such NPs promoted permeation of encapsulated CE at a CE polyphenolic concentration of at least 2 µg GAE/mL. More mucoadhesive NPs from Ch-der, coded quaternary ammonium S-protected thiolated chitosan (QA-Ch-S-pro) NP, promoted CE GI permeation of 0.5 µg GAE/mL. At higher concentrations of Ch-der polymers, the resulting NPs containing CE were toxic toward Caco-2 and HT29-MTX cells. CE protected human umbilical vein endothelial cells (HUVECs) from oxidative stress and maintained its activity when entrapped in PLGA NPs. CE encapsulated in QA-Ch-S-pro NP protected HUVECs from oxidative stress, even more effectively than non-encapsulated CE. Furthermore, mucoadhesive NPs from Ch-der were more effective antioxidant protectors than PLGA NPs, but less cytotoxic PLGA NPs could be more useful when comparatively high therapeutic antioxidant doses are needed.

## 1. Introduction

Among agri-food products, sweet cherries are widely described for their nutritional properties and the beneficial effects of their compounds on human health [1,2,3,4]. Indeed, polyphenols contained in cherries, such as flavonoids and anthocyanins, are endowed with antioxidant and anti-inflammatory properties [5,6,7,8]. Among flavonoids, catechins recently showed the best antioxidant properties when compared with other antioxidants [9].

In previous work [10], we studied the antioxidant ability of a particular strain of cherries, i.e., Crognola Capannile from sweet cherry (*Prunus avium* L.), which is one of the more ancient autochthonous varieties found in Tuscany, Italy. This variety best adapted itself to the territory where it grows; therefore, it shows better nutraceutical characteristics than non-autochthonous varieties [11,12,13]. We chose the autochthonous variety of *Prunus avium* L. cherry since we aimed at protecting the agrobiodiversity of our regional territory. In particular, we chose the Crognola Capannile among six *Prunus avium* L. varieties, because it has the highest content of antioxidant polyphenols, as shown in previous papers [11,13]; therefore, it was considered the most relevant to the purposes of the present work. The encapsulation of cherry extracts (CEs) in polymeric nanoparticles (NPs) prepared with two different derivatives of quaternary ammonium chitosan (QA-Ch) can protect CE from the degradation in the gastrointestinal (GI) tract following non-protected oral administration. In fact, such a degradation is the cause of the low bioavailability of antioxidants extracted from food. The abovementioned NPs showed the ability to promote CE transport across intestinal epithelium, while only CE encapsulated in NPs made from QA-Ch derivatives containing protected thiol groups (QA-Ch-S-pro) could improve the resistance of endothelial cells to oxidative stress compared to non-encapsulated CE.

The previous findings suggest that the basic polymer from which NPs are prepared may be relevant to CE protection from degradation, promotion of CE intestinal absorption, protection of endothelial cells from oxidative stress [14], and NP properties such as size, zeta potential, and mucoadhesivity.

Poly(lactic-*co*-glycolic acid) (PLGA) is another polymer that is widely and successfully used to encapsulate extracts of natural products via different techniques. PLGA is a synthetic polymer approved by the United States (US) Food and Drug Administration (FDA) and European Medicine Agency (EMA) for the production of polymeric biocompatible and biodegradable NPs encapsulating polyphenol-rich fruits and other nutraceuticals [15,16,17,18,19].

However, experimental data are still lacking that allow evaluation and comparison between NPs from QA-Ch derivatives and PLGA NPs on the basis of the nutraceutical effectiveness of the encapsulated CE. Making such an evaluation and comparison is the purpose of the present work. Some of the relevant data for NP from QA-Ch derivatives were taken from our previous work [10]. In the present study, PLGA NPs were prepared with a double emulsion technique (w/o/w) which showed promise of satisfactory physical characteristics of the resulting NPs [20]. Moreover, the capacity of PLGA to decrease cytotoxicity [21] could allow the encapsulation of high CE concentrations in NP, which was not possible with Ch-derivative (Ch-der) NPs. Finally, the CE-containing PLGA NPs were compared with the NPs based on QA-Ch derivatives for the ability to promote the permeation of CE through the GI epithelium, using a triple-cell co-culture model (Caco-2/HT29-MTX/Raji B) [22], and to protect the human umbilical vein endothelial cells (HUVECs) from oxidative stress.

## 2. Results and Discussion

### 2.1. Nanoparticle Characterization

The characteristics of PLGA NPs, such as size, zeta potential (ZP), and entrapment efficiency (EE), determined via both a direct and indirect method (described in Materials and Methods) are shown in Table 1. Data show that neither particle size (about 200 nm) nor zeta potential (about −10 mV) depend on the CE concentration used to prepare the NPs. However, with the exception of the lowest CE concentration (5 mg/mL, 100 µg gallic acid equivalent (GAE)/mL), all the other concentrations tested always yielded EEs >50% via the indirect method. In fact, using the indirect method, we observed an increase in EE following an increase in polyphenol concentration. Conversely, using the direct method, we observed the lowest EE with the intermediate polyphenol concentration. It can also be seen that all EE values obtained via the direct method were lower than the corresponding ones obtained via the indirect method. This may be due to the direct method being less reliable than the indirect one, perhaps because the disruption of nanoparticles, required by the direct method, was not complete. Nevertheless, the EE at the highest concentration of entrapped CE (840 µg GAE/mL) showed the promising value of 80% for both methods. 

It should be pointed out that the particle size of PLGA NPs listed in Table 1 is significantly smaller than that of QA-Ch NP and QA-Ch-S-pro NPs, previously found to be about 350 nm [10]. A further difference resides in the zeta potential of particles, which is negative in the present cases, whereas it was found to be positive with the QA-Ch NP and QA-Ch-S-pro NPs because of quaternary ammonium residues on the NP surface [10].

**Figure 1:**
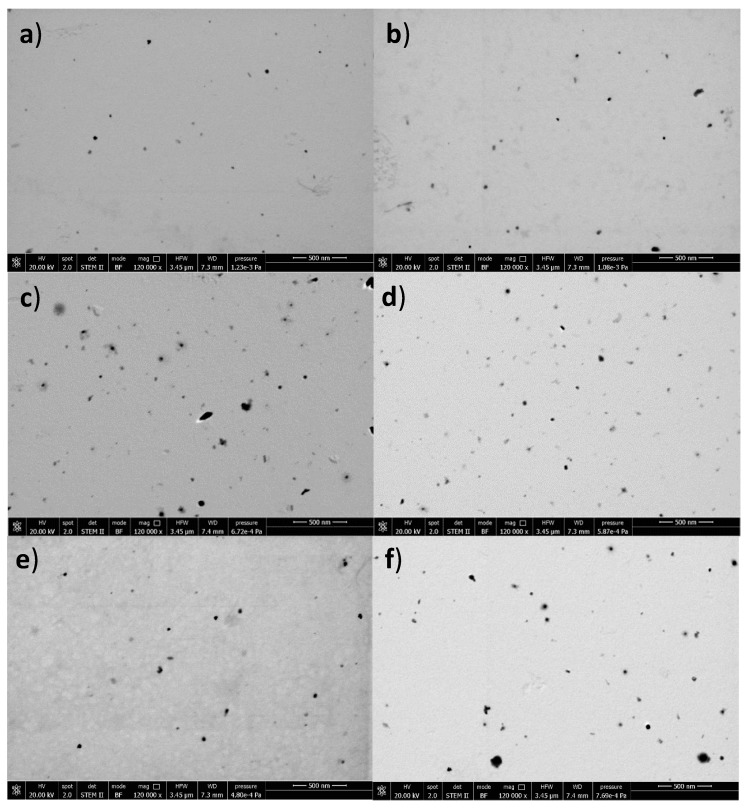
Scanning transmission electron microscopy (STEM) photos of poly(lactic-*co*-glycolic acid) (PLGA) nanoparticles (NPs) (**a**,**b**), quaternary ammonium chitosan (QA-Ch) NPs (**c**,**d**) and quaternary ammonium S-protected thiolated chitosan (QA-Ch-S-pro) NPs (**e**,**f**) obtained using a field-emission scanning electron microscope with a resolution of 120,000× and a scale bar of 500 nm. Photos (**a**,**c**,**e**) on the left represent the morphological analysis of empty NPs; photos (**b**,**d**,**f**) on the right represent the respective cheery extract (CE)-loaded NPs.

The scanning transmission electron microscopy (STEM) photos of the nanoparticles reported in Figure 1 show that the dimensions of the PLGA NPs are smaller than those of QA-Ch NP and QA-Ch-S-pro NPs, and that there is no difference between the empty NPs and the respective CE-loaded NPs. These data are in agreement with those obtained by dynamic light scattering. In the photos, some aggregates are visible, probably due to the centrifugation of the sample and subsequent re-suspension of the pellet in water before the analysis.

### 2.2. Ex Vivo Mucoadhesivity Studies

It was previously shown that the NP mucoadhesivity can both markedly affect the NP ability to cross the mucus layer that covers the GI [23], and promote the absorption of the active contents encapsulated in the NPs [24,25]. For this purpose, the mucoadhesivity of the NP was determined, and its effect on intestinal permeation was tested and compared with that of the basic non-aggregated polymers in solution. The polymer fraction adsorbed onto the intestinal tissue calculated on the basis of the initial polymer amount used can be found in Table 2. The following rank order of NP mucoadhesivity was observed: QA-Ch-S-pro NPs > QA-Ch NPs > PLGA NPs. The same order was seen for non-aggregated polymers in solution, although PLGA, which is not water-soluble, could not be tested. It should be stressed that the NPs appeared more mucoadhesive than the respective constituent polymers, probably because of NP entrapment in the aqueous pores of mucus gel. Unlike the case of NPs, with non-aggregated polymers, the equilibrium between polymer entrapped in mucus and polymer free in solution may be reversible.

### 2.3. Cell Viability

**Figure 2:**
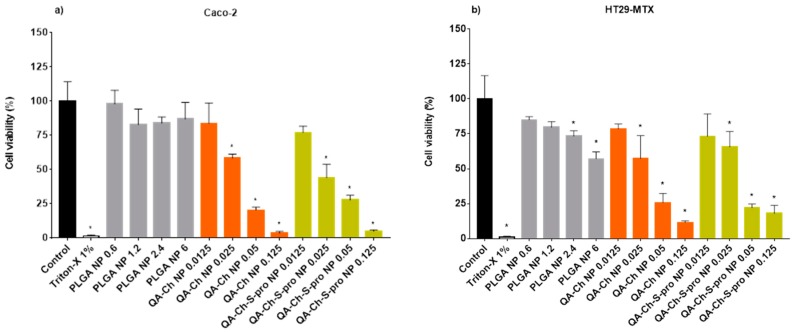
Caco-2 (**a**) and HT29-MTX (**b**) cell viability after 24-h treatment with empty PLGA NPs (0.6–6 mg PLGA/mL culture medium) or NPs from Ch derivatives (0.0125–0.125 mg QA-Ch or QA-Ch-S-pro/mL culture medium). Triton-X 1% was used as a negative control. *, significantly different from control (cells with medium).

The viability of Caco-2 and HT29-MTX human colon carcinoma cells, following 24 h of incubation with empty PLGA NPs, QA-Ch NPs, or QA-Ch-S-pro NPs, is reported in Figure 2a,b, respectively.

**Figure 3:**
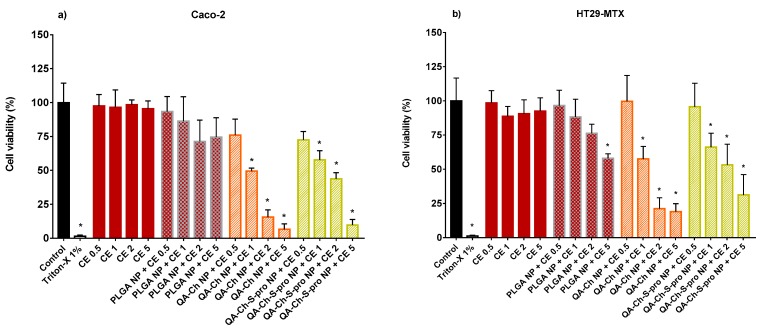
Caco-2 (**a**) and HT29-MTX (**b**) cell viability after 24-h treatment with CE free or loaded in different types of NPs at the polyphenol concentrations of 0.5, 1, 2, and 5 µg/mL gallic acid equivalent (GAE). Triton-X 1% was used as a negative control. *, significantly different from control.

The data appearing in Figure 1a show that PLGA NPs are not toxic toward Caco-2 cells, regardless of the concentration tested. Conversely, the data reported in Figure 2b show a viability reduction of HT29-MTX at a PLGA NP concentration as low as 2.4 mg/mL. Instead, the NPs based on Ch derivatives were found to be toxic starting from the concentration of 0.025 mg/mL with no significant difference between the two NP types studied. Data then show that PLGA NPs are less toxic toward cells than the NPs based on Ch derivatives. The concentrations of empty PLGA NPs, QA-Ch NPs, and QA-Ch-S-pro NPs tested correspond exactly to the respective CE-loaded NPs, the relevant data of which are shown in Figure 3a,b.

It is noteworthy that the NPs based on the Ch derivatives have higher EE values than those based on PLGA [10]; therefore, a lower polymer amount is enough to entrap the same CE amount as the PLGA NPs. The viability of Caco-2 and HT29-MTX cells following incubation with CE, free or entrapped in PLGA NPs, QA-Ch NPs, or QA-Ch-S-pro NPs, is shown in Figure 3a,b. Figure 3a shows that the viability of Caco-2 cells is not significantly reduced by CE, whether free or encapsulated in PLGA NPs, whereas it is reduced by CE encapsulated in QA-Ch NPs or in QA-Ch-S-pro NPs, starting from a CE concentration as low as 1 µg GAE/mL. On the other hand, a significant reduction in HT29-MTX cell viability for CE encapsulated in PLGA NPs is observed in Figure 3b, starting from a CE concentration of 5 µg GAE/mL, while the non-encapsulated CE showed no toxicity regardless of the concentrations tested. Instead, the CE encapsulated in QA-Ch NPs or QA-Ch-S-pro NPs showed toxicity toward HT29-MTX cells starting from the CE concentration of 1 µg GAE/mL. In fact, considering a cell viability higher than 70% as acceptable (ISO 10993-5: 2009(E)), we carried out the subsequent experiments with the PLGA NPs loaded with CE up to the concentration of 2 µg GAE/mL, and the QA-Ch NPs and QA-Ch-S-pro NPs loaded with CE at the concentration of 0.5 µg GAE/mL.

### 2.4. Permeability Studies in a Triple Co-Culture Intestine Cell Model

The triple co-culture model, Caco-2/HT29-MTX/Raji B, was used for the permeation experiments as it mimics the human intestine better than the Caco-2 model. Indeed, the HT29-MTX cells are mucus-secreting cells and the Raji B cells can induce differentiation of the phenotype Caco-2 into M cells [26]. 

**Figure 4:**
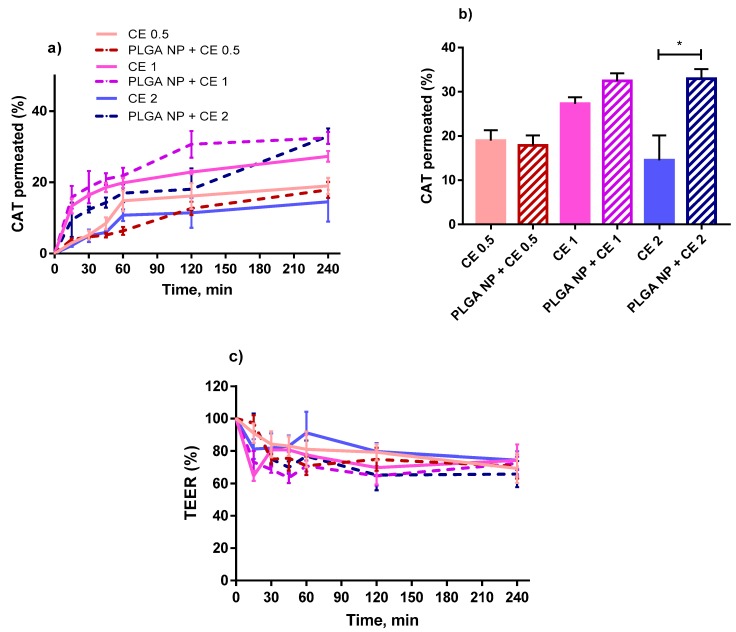
Percentage of catechin (CAT) permeated (**a**) from non-encapsulated CE or CE-loaded PLGA NP at the CE polyphenol concentrations of 0.5, 1, or 2 µg/mL GAE and corresponding transepithelial electrical resistance (TEER) values (**c**) during the permeability study in the triple-cell co-culture model (Caco-2/HT29-MTX/Raji B). (**b**) represents the % CAT permeated after 240 min. *, significantly different from each other.

Figure 4 represents permeation data for CE either free or encapsulated in PLGA NPs at different concentrations (Figure 4a,b), and corresponding transepithelial electrical resistance (TEER) values measured during the permeation experiments (Figure 4c). The TEER value before the experiment was always around 500 Ω·cm^2^. Based on TEER value, the GI epithelia are classified as “tight” (TEER of about 2000 Ω·cm^2^), “intermediate” (TEER of 300–400 Ω·cm^2^), and “leaky” (TEER of 50–100 Ω·cm^2^) [27].

In Figure 4c, no significant difference in TEER is observed between the dispersions tested, and, in all cases, the TEER, although decreasing in time, never drops to less than 70% of the initial value. Such a decrease is compatible with an acceptable integrity of the epithelial barrier [28] and testifies to the reliability of data represented in Figure 4a,b. These results indicate a significant promotion of permeation by PLGA NPs only with the highest concentration tested, i.e., a 2.2-fold increase in catechin (CAT) permeated over the whole time of experiment (240 min) compared to the respective control (non-encapsulated CE). In fact, others also found the ability of PLGA NPs to promote the permeation of encapsulated drugs and ascribed it to the hydrophobic nature of PLGA NPs, which could promote the NP hydrophobic interaction with the cell lipid double layer [29]. 

**Figure 5:**
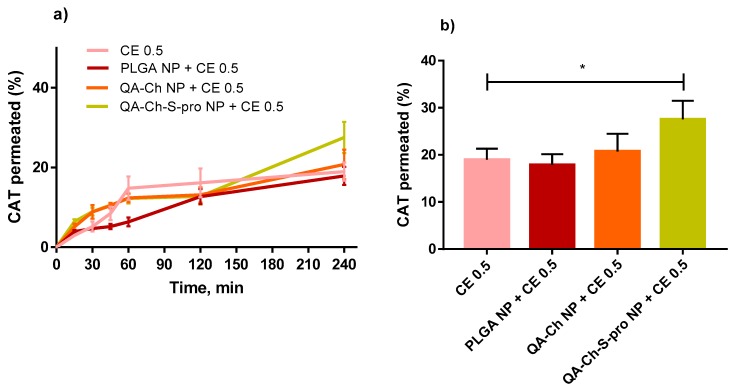
Comparison between PLGA NPs and Ch-derivative (QA-Ch and QA-Ch-S-pro) NPs loaded with CE for CE permeation properties. (**a**) represents % CAT permeated from non-encapsulated CE or CE-loaded NPs at the CE concentration of 0.5 µg/mL GAE, over time. (**b**) represents the % CAT contained in CE 0.5 µg/mL GAE, permeated after 240 min. *, significantly different from each other.

Interestingly, the CAT percentage permeated with PLGA NPs + CE 1 µg GAE/mL is not significantly different from that permeated with PLGA NPs + CE 2 µg GAE/mL, thus indicating that, above a given concentration in donor phase, the CE does not influence the CAT % if encapsulated in NPs. Even a reduction in CAT % with increased non-encapsulated CE dose in the donor phase was observed. This could be explained considering that CE that is not encapsulated in NPs is less stable in solution than the encapsulated one, as it was found in our previous work [10]. Therefore, an increase in initial concentration is not reflected in a higher undegraded CAT concentration, available for permeation. Permeation data for PLGA NPs and more or less mucoadhesive NPs based on chitosan derivatives, i.e., QA-Ch-S-pro NP (more mucoadhesive) or QA-Ch NP (less mucoadhesive) [10], all containing the same CE concentration (0.5 µg GAE/mL), are compared in Figure 5. In all these cases, the TEER values were not significantly different from those reported in Figure 4c (data not shown). In Figure 5a, it is observed that only the NPs based on QA-Ch-S-pro significantly promoted the permeation of the encapsulated CE. This can be justified well by the triple co-culture model used in the permeation studies containing mucus-secreting cells [22] that can put into evidence the possible permeability differences ascribable to NP mucoadhesivity. Indeed, a stronger NP adhesion to the mucus layer can allow the permeant to reside at the permeation site for longer than the less mucoadhesive NPs. From the profile of the curves in Figure 4a and Figure 5a, a curvature is seen after 60 min that is typical of fan endocytosis-mediated internalization [30].

### 2.5. Protective Effect of Non-Encapsulated CE or NP-Loaded CE from Oxidative Stress

**Figure 6:**
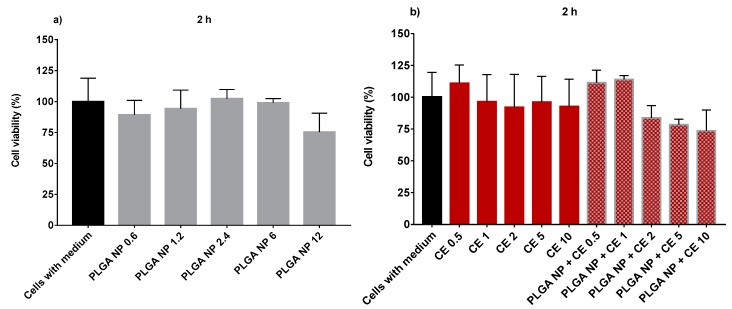
Human umbilical vein endothelial cell (HUVEC) viability after 2-h treatment with empty PLGA NPs up to 12 mg/mL (**a**), non-encapsulated CE, and CE-loaded NPs (**b**) at different CE concentrations (0.5–10 µg/mL GAE).

**Figure 7:**
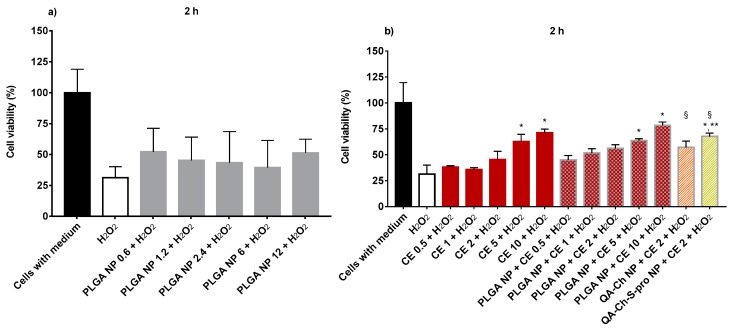
HUVEC viability after 2-h treatment with empty PLGA NPs (**a**), non-encapsulated CE, and CE-loaded NPs (**b**) and the subsequent 1-h treatment with 50 μM H_2_O_2_. Figure 6b also shows a comparison between PLGA NPs and NPs from Ch derivatives (QA-Ch and QA-Ch-S-pro) for HUVEC protection from H_2_O_2_-induced oxidative stress after 2-h treatments. *, significantly different from negative control (H_2_O_2_); **, significantly different from CE 2 + H_2_O_2_, PLGA NPs + CE 2 + H_2_O_2_, and QA-Ch NPs + CE 2 + H_2_O_2_; §, data taken from Reference [10].

Before studying the protective effect of empty PLGA NPs or CE, free or encapsulated in PLGA NPs, from oxidative stress, their cytotoxicity on HUVECs following two hours of incubation was evaluated. The relevant values for increasing concentrations of empty PLGA NPs in the 0.6–12 mg/mL, reported in Figure 6a, show the absence of any toxicity of the concentrations tested. These concentrations are exactly the same as those of the CE-loaded PLGA NPs, the relevant data of which are shown in Figure 6b. In this figure, it is also shown that CEs, whether free or encapsulated in PLGA NPs, are not toxic on HUVECs at any of the concentrations tested. Therefore, the protection effect from oxidative stress was evaluated at all concentrations tested, as reported in Figure 6a,b. Data for the ability of empty PLGA NPs or CE either free or encapsulated in PLGA NPs to protect HUVECs from oxidative stress induced by H_2_O_2_ are found in Figure 7a,b. The H_2_O_2_ toxic effect on HUVECs is demonstrated by a reduction in cell viability until about 30%. Data reported in Figure 7a show that no protective antioxidant effect is exerted by 2-h pre-treatment of HUVECs with empty PLGA NPs. Instead, data in Figure 7b show that non-encapsulated CEs exert a significant effect against HUVEC oxidation at 5 and 10 µg GAE/mL concentrations. The same effect is observed with HUVEC pre-treated for 2 h with CE encapsulated in PLGA NPs at 5 or 10 µg GAE/mL. Data indicate that the CEs encapsulated in PLGA NPs maintain their antioxidant activity. Figure 7b also shows the results obtained in our previous work [10] with CE encapsulated in QA-Ch NPs and QA-Ch-S-pro NPs. In the figure, the ability of CE encapsulated in QA-Ch-S-pro NPs to protect the HUVECs from oxidative stress is seen even at the CE concentration of 2 µg GAE/mL, which was ineffective with the CE either free or encapsulated in PLGA NPs. This result can be ascribed to a synergistic antioxidant effect of CE and the protected pendant SH groups on the surface of the QA-Ch-S-pro NPs that were not present on the QA-Ch NP surface. The latter were indeed unable to protect HUVECs from oxidative stress. In fact, the protected thiols are known to act as strong reducing groups [31]. The enzyme protein disulfide isomerase catalyzes the formation or rupture of disulfide bonds, i.e., redox reactions where thiol is in the reduced (and reductive) form.

## 3. Materials and Methods

For the preparation of the PLGA NPs, PLGA 5004 A (50:50, molecular weight (MW) ≈ 44 kDa), was generously offered by Corbion-Purac Biomaterials (Netherlands). Ethyl acetate (EA), Kolliphor^®^ P 407 and fluorescein isothiocyanate (FITC) were purchased from Sigma-Aldrich (St. Louis, MO, USA). The cherry extracts were obtained from a Tuscan cherry variety of *Prunus avium* L. (sweet cherry), Crognola Capannile, as already described [10]. The total polyphenol content (TPC) of CE samples, expressed as gallic acid equivalent (GAE), was 26.7 µg/mL GAE per mg of dry weight, while the total antioxidant potential of CE, determined using the ferric-reducing antioxidant power (FRAP) assay, was 0.229 mg of Fe^2+^/mL. The following polymer derivatives were prepared according to the methods described in the references cited: reduced-MW hyaluronic acid rHA, viscosimetric MW 470 kDa, from HA MW 950 kDa (Contipro, Dolní Dobrouč, Czech Republic) [32]; quaternary ammonium chitosan (QA-Ch) conjugates synthesized at 60 °C, from low-molecular-weight Ch (Sigma) [33]; thiolated derivative of QA-Ch [34] and its derivative with protected thiols, coded QA-Ch-S-pro [24]. The following chemicals for the HPLC analysis were purchased from Sigma-Aldrich: phosphoric acid 85% *w*/*v*, methanol, and (+)-catechin hydrate >98% pure as a standard. The following reagents for cells culture were purchased from Sigma: M199 medium, fetal bovine serum (FBS), heparin sodium, gelatin solution 2%, endothelial cell growth supplement (ECGS), 3-(4,5-dimethylthiazol-2-yl)-2,5-diphenyltetrazolium bromide (MTT), and dimethyl sulfoxide (DMSO). Dulbecco’s modified Eagle’s medium (DMEM) with 4.5 g/L glucose from Lonza, Hank’s balanced salt solution (HBSS 1× without phenol red), penicillin–streptomycin mixture (Pen-Strep) from Gibco (Waltham, MA, USA), and Triton X-100 from Spi-Chem (Atlanta, GA, USA) were also used for in vitro studies.

### 3.1. Catechin Determination

For the quantification of catechins (CATs) in CE, high-performance liquid chromatography (HPLC) as described by Nunes et al. [35] was adapted. An RP-C18 *LiChroCART*^®^ column (250 × 4.6; 5 µM) and a mobile phase consisting of methanol–phosphoric acid 0.01 M (15:85, *v*/*v*) were used. The eluent flow rate was 0.8 mL/min. The temperature of the column was maintained at 40 °C, and the injection volume was 20 μL. Ultraviolet detection was set at 230 nm, and the total area of peak was used to quantify the CAT present in CE, based on a CAT calibration curve. Catechins in CE were 18.52 μg/mg of dry weight, which was about 70% of CE TPC.

### 3.2. Preparation and Characterization of PLGA NPs

CE-loaded PLGA NPs were prepared through a w/o/w double emulsion technique [20]. One hundred milligrams of PLGA 50:50 was dissolved in 2 mL of EA overnight. Then, 100 μL of different concentrations of CE solution in water (5, 10, 15, and 30 mg/mL), corresponding to polyphenol concentrations of 100, 250, 420, and 840 μg/mL GAE, was added. The first emulsion was obtained after 1.5 min of vortexing. To stabilize this emulsion, 4 mL of the surfactant solution, 0.5 % Kolliphor^®^ P 407 in water, was added, then the emulsion was homogenized for 1 min with a Vibra-Cell™ ultrasonic processor (amplitude, 70%). Finally, the second emulsion (w/o/w) was added into 8 mL of the same surfactant solution. This emulsion was left for 3 h in a fume hood under magnetic stirring at 300 rpm for EA evaporation. The PLGA NP formulations obtained were washed with Milli-Q^®^ water (Millipore, Toronto, CN, USA) followed by centrifugation at 4°C and 600 g in Amicon^®^ Ultra-15, MW cutoff (MWCO) 30-kDa filters (Merck Millipore, Toronto, CN, USA). The washing was repeated three times. The same above procedure was used to produce plain NPs. After production, each NP suspension was characterized for average particles size, polydispersity index, and zeta potential (ZP) by dynamic light scattering, using a Malvern Zetasizer Nano ZS instrument (Malvern Instruments Ltd.,Worcestershire, UK). For the measurement, a set of three replicate samples was diluted with sodium chloride to an appropriate concentration. The PLGA NPs obtained after washing were freeze-dried and the powder was weighed to evaluate the amount of PLGA involved in the NP formation. 

The entrapment efficiency (EE) of CE in PLGA NPs was determined via both a direct and an indirect method. According to the direct method, freeze-dried PLGA NPs (10 mg) were destroyed by the addition of 5 mL of dichloromethane. After NP complete dissolution, a liquid–liquid extraction (1 mL H_2_O) was performed in order to quantify CE CAT in the aqueous phase (CAT_aq_). The collected solution was then analyzed by HPLC for CAT as previously described. Following the indirect method, the amount of CAT in CE-loaded NPs was calculated from the difference between the total amount of CAT (CAT_tot_) used to prepare the NP dispersion and the CAT determined in the supernatant after centrifugation of the dispersion (CAT_sup_). The EE percentages obtained via the direct and the indirect method were calculated from the following equations:

EE % (direct method) = [CAT_aq_/CAT_tot_] × 100; (1)

EE % (indirect method) = [(CAT_tot_ − CAT_sup_)/CAT_tot_] × 100. (2)

Empty and CE-loaded NP morphology was investigated by means of scanning transmission electron microscopy (STEM, FEI Company, Hillsboro, OR, USA), using a field-emission scanning electron microscope (FE-SEM, FEI Quanta 450 ESEM FEG, FEI Company, Hillsboro, OR, USA). After preparation, all the PLGA and Ch-der NPs were purified by centrifugation (20 min at 8000 rpm and 4 °C, Thermo Scientific MTX 150 (Langenselbold, Germany) and the resulting pellets were re-suspended in deionized water, in 2 mL and 500 µL, respectively. Then, all the PLGA and Ch-der NP samples were diluted 1:100 and 1:10, respectively, in ethanol and added on the STEM-mesh copper grid.

### 3.3. FITC Labeling of QA-Ch, QA-Ch-S-pro, and PLGA

A previously described procedure was followed to obtain the FITC-labeled QA-Ch and QA-Ch-S-pro [32].

For the labeling of PLGA, a solution of FITC in dimethyl sulfoxide (0.5 mL, 2mg/mL) was added to a solution of PLGA in dimethyl sulfoxide (1.5 mL, 67 mg/mL) and the mixture was incubated for 12–16 h at 4 °C. Thirty milliliters of deionized water was added to the solution, which caused PLGA precipitation. The resulting system was centrifuged for 10 min at 4000 rpm to clear the labeled polymer of non-reacted FITC, and dried for 12 h at 37 °C in a vacuum oven.

### 3.4. Ex Vivo Mucoadhesivity Studies

A previously described procedure was followed [32]. Organ harvesting from rats was authorized by the animal welfare organization of the University of Pisa on 4 April 2017 (protocol number 16746/2017). The intestinal mucosa was excised from non-fasting male Wistar rats weighing 250–300 g. After sacrificing the rats, the first 50 cm of small intestine was immediately removed. Four 10-cm segments were cut out of the excised intestine, opened by longitudinal cutting, and the mucosa WAS gently rinsed free of luminal content. Each segment was then immersed in 1 mL of FITC-labeled PLGA NPS, QA-Ch NPS, or QA-Ch-S-pro NPS. Incubation at 37 °C, under bubbling of a mixture of 95% O_2_ and 5% of CO_2_, lasted 4 h with no damage to the mucosa [34]. Then, 300 μL was withdrawn, appropriately diluted, and analyzed fluorometrically with reference to the relative calibration curve. The adsorbed mass fraction was calculated from the difference between the concentrations of FITC-labeled materials determined before and after incubation.

### 3.5. Cell Viability Assay on Caco-2 and HT29-MTX

An MTT assay was performed on a Caco-2 clone (C2BBe1) from the American Typical Culture Collection (ATCC, Wesel, Germany) and HT29-MTX human colon carcinoma cells kindly provided by Dr T. Lesuffleur (INSERM U178, Villejuif, France) to evaluate cell viability after CE and NP treatments. Monocultures of both Caco-2 and HT29-MTX were seeded at densities of 2 × 10^4^ and 1 × 10^4^ cells/cm^2^, respectively, in DMEM complete (10% fetal bovine serum (FBS), 1% antibiotic penicillin–streptomycin (Pen/Strep), 1% non-essential amino acids (NEAA)). After 24 h of incubation, cells were treated with different concentrations of empty PLGA NPs (0.6, 1.2, 2.4, and 6 mg PLGA/mL culture medium) or NPs based on QA-Ch derivatives (0.0125, 0.025, 0.05, and 0.125 mg of QA-Ch/mL of culture medium or the same concentrations of QA-Ch-S-pro in culture medium) [10]. Moreover, cells were treated with PLGA NPs or NPs based on QA-Ch derivatives loaded with different CE concentrations (0.5, 1, 2, and 5 μg GAE/mL culture medium). All types of CE-loaded NPs were prepared by dilution of the NP dispersion containing 30 mg/mL CE (840 μg GAE/mL). CE solutions at the same polyphenol concentrations (0.5–5 μg GAE/mL culture medium) were used as the respective controls. DMEM without FBS was used as culture medium for all the treatments. After 24 h of treatment, the medium was removed, and 200 μL of 0.5 mg/mL MTT solution was added to each well, followed by incubation of the plates at 37 °C for 4 h. The reaction was terminated by removal of MTT solution and addition of 200 μL of dimethyl sulfoxide. The absorbance of reduced MTT was measured at 590 nm and 630 nm using a microtiter plate reader (Biotek-Synergy H1 Hibrid reader, Gen5 2.01 program). The final results were expressed as the percentage of cell viability on a control basis (cells with medium).

### 3.6. Permeability Studies in a Triple-Cell Co-Culture Model

To predict the oral permeability of CE and CE-loaded NPs, a triple-cell co-culture model (Caco2/HT29-MTX/Raji B) was prepared using a method previously described and validated [22,26,36].

Briefly, Caco-2 and HT29-MTX cells were seeded in Millicell Hanging Cell Culture Insert, PET 1 µM, six-well (Merck Millipore) at density of 1 × 10^5^ cells/cm^2^ in the proportion of 90:10; the Raji B cell (from ATCC, Manassas, VA, USA) suspension was seeded on the 14th day in the basolateral compartment at a density of 1 × 10^5^ cells/cm^2^. Then, cells were maintained in culture for another seven days. DMEM complete was used both in the apical (1.5 mL) and the basolateral (2.5 mL) side. TEER (transepithelial electrical resistance) was measured after eight days of co-culture and then every 2–3 days before changing medium, up to the day of the experiment. After Raji B seeding, the medium in the basolateral compartment was no longer changed. At day 21, the dispersions of PLGA NPs loaded with different CE concentrations (0.5, 1, and 2 μg GAE/mL HBSS), or QA-Ch NPs and QA-Ch-S-pro NPs loaded with the CE concentration of 0.5 μg GAE/mL HBSS were tested for the CE CAT permeability. Cherry extract solutions at the same polyphenol concentrations were used as the respective controls. All the formulations were put in the apical side. Samples were withdrawn from the basolateral side at different time points: 15, 30, 45, 60, 120, and 240 min. For the permeation studies, the TEER was measured before starting and before each sample withdrawal over the whole duration of experiment. CE CAT permeated over time was determined by HPLC analysis, as described in Section 3.1, and reported as a percentage of CAT permeated.

### 3.7. Effect of Empty NPs or CE-Loaded NPs on HUVEC Viability

HUVECs were provided by ScienCell (Carlsbad, CA, USA). They were incubated for 2 h or 24 h with dispersions of empty PLGA NPs (0.6, 1.2, 2.4, 6, and 12 mg PLGA/mL culture medium), or PLGA NPs loaded with different CE concentrations (0.5, 1, 2, 5, and 10 μg GAE/mL culture medium) prepared by dilution of the dispersion containing 840 μg GAE/mL. Cherry extract solutions having the same polyphenol concentrations as the CE-loaded NPs were used as the respective controls. Then, cells were treated with 50 µM commercial H_2_O_2_ for 1 h to induce oxidative stress, as previously described [10]. All solutions/dispersions were prepared in M199 5% FBS. Cells treated only with medium were used as a positive control. MTT assay was carried out at the end of the treatments to evaluate HUVEC viability as described in Section 3.5.

### 3.8. Statistical Analysis

The GraphPad Prism Software vs. 7.0 (GraphPad Software Inc., La Jolla, CA, USA) was used for the statistical analysis of data. All results were presented as means ± standard deviation of at least three independent experiments. The difference between values was evaluated by the Student’s *t*-test. Differences were considered significant, i.e., the null hypothesis was rejected, for *p*-values lower than 0.05.

## 4. Conclusions

Nanoparticles based on PLGA, able to promote permeability of encapsulated CE at the concentration of 2 µg GAE/mL, were successfully prepared. The QA-Ch-S-pro NPs, prepared in a previous work, showed the ability to promote transepithelial CE permeation even at the lowest concentration of 0.5 µg GAE/mL. However, at higher concentrations, they were found to be toxic toward either Caco-2 or HT29-MTX. The antioxidant activity of non-encapsulated CE was maintained with the CE encapsulated in PLGA NPs, while the QA-Ch-S-pro NPs showed antioxidant activity even at CE concentrations where non-encapsulated CE was ineffective, thanks to the intrinsic antioxidant activity of QA-Ch-S-pro. The above results lead to the conclusion that, although QA-Ch-S-pro NPs are more effective than PLGA NPs, the cytotoxicity of the former may put a limit on its use in those cases where comparatively high doses are required. In these cases, the use of PLGA NPs, which showed low cytotoxicity and could allow administration of higher CE doses, could be more convenient.

## Figures and Tables

**Table 1:** Characteristics of poly(lactic-*co*-glycolic acid) (PLGA) nanoparticles (NPs) loaded with different cherry extract (CE) polyphenol concentrations, (100–840 µg gallic acid equivalent (GAE)/mL). Entrapment efficiency (EE%) was obtained via a direct or indirect method and the HPLC analysis of CE catechins.

NP Type	Nanoparticle Size, nm (Polydispersity Index)	Zeta Potential (ZP), mV	EE % (Direct Method)	EE % (Indirect Method)
PLGA NP + CE 100	216.0 ± 2.6 (0.06 ± 0.03)	−11.0 ± 1.23	-	-
PLGA NP + CE 250	216.8 ± 4.9 (0.06 ± 0.02)	−10.9 ± 1.04	64.8 ± 2.3	51.7 ± 8.9
PLGA NP + CE 420	208.4 ± 4.9 (0.05 ± 0.02)	−12.6 ± 0.87	43.9 ± 4.6	70.8 ± 5.5
PLGA NP + CE 840	206.1 ± 1.8 (0.06 ± 0.03)	−8.36 ± 1.07	79.8 ± 6.2	88.6 ± 6.2

**Table 2:** Mass fraction adsorbed onto rat intestinal mucosa from solutions of chitosan derivative (QA-Ch or QA-Ch-S-pro), or dispersions of PLGA NPs, QA-Ch NPs, or QA-Ch-S-pro NPs. PLGA adsorption was not determined because of its insolubility in water.

Formulation	Adsorbed Mass Fraction, %
QA-Ch	23.5 ± 3.1 ^a,b^
QA-Ch-S-pro	40.7 ± 2.0 ^b,c^
PLGA	ND
QA-Ch NP	39.7 ± 4.6 ^a,d^
QA-Ch-S-pro NP	80.5 ± 6.4 ^c,d^
PLGA NP	-

QA-Ch = quaternary ammonium chitosan; QA-Ch-S-pro = quaternary ammonium S-protected thiolated chitosan; PLGA = poly(lactic-*co*-glycolic acid); ND = not determined. ^a,b,c,d^, significantly different from each other.
